# Tinnitus: Distinguishing between Subjectively Perceived Loudness and Tinnitus-Related Distress

**DOI:** 10.1371/journal.pone.0034583

**Published:** 2012-04-18

**Authors:** Elisabeth Wallhäusser-Franke, Joachim Brade, Tobias Balkenhol, Roberto D'Amelio, Andrea Seegmüller, Wolfgang Delb

**Affiliations:** 1 Department of Phoniatrics and Audiology, Medical Faculty Mannheim, Heidelberg University, Mannheim, Germany; 2 Department of Medical Statistics and Biomathematics, Medical Faculty Mannheim, Heidelberg University, Mannheim, Germany; 3 Department of Internal Medicine IV and Neurocenter, University Clinic, Saarland University, Homburg/Saar, Germany; University of Regensburg, Germany

## Abstract

**Objectives:**

Overall success of current tinnitus therapies is low, which may be due to the heterogeneity of tinnitus patients. Therefore, subclassification of tinnitus patients is expected to improve therapeutic allocation, which, in turn, is hoped to improve therapeutic success for the individual patient. The present study aims to define factors that differentially influence subjectively perceived tinnitus loudness and tinnitus-related distress.

**Methods:**

In a questionnaire-based cross-sectional survey, the data of 4705 individuals with tinnitus were analyzed. The self-report questionnaire contained items about subjective tinnitus loudness, type of onset, awareness and localization of the tinnitus, hearing impairment, chronic comorbidities, sleep quality, and psychometrically validated questionnaires addressing tinnitus-related distress, depressivity, anxiety, and somatic symptom severity. In a binary step-wise logistic regression model, we tested the predictive power of these variables on subjective tinnitus loudness and tinnitus-related distress.

**Results:**

The present data contribute to the distinction between subjective tinnitus loudness and tinnitus-related distress. Whereas subjective loudness was associated with permanent awareness and binaural localization of the tinnitus, tinnitus-related distress was associated with depressivity, anxiety, and somatic symptom severity.

**Conclusions:**

Subjective tinnitus loudness and the potential presence of severe depressivity, anxiety, and somatic symptom severity should be assessed separately from tinnitus-related distress. If loud tinnitus is the major complaint together with mild or moderate tinnitus-related distress, therapies should focus on auditory perception. If levels of depressivity, anxiety or somatic symptom severity are severe, therapies and further diagnosis should focus on these symptoms at first.

## Introduction

Subjective tinnitus is a sound that does not originate from an external or body sound source and that is heard only by those affected. Tinnitus is a widespread symptom with 30–40% of the adult population experiencing tinnitus during their life, and 0.5–2.5% being severely affected by a tinnitus that interferes with life quality [1]–[4]. The majority of tinnitus patients are hearing impaired [4], and many additionally express hypersensitivity to environmental sounds [5]. Treatment of hearing loss by hearing aids or cochlear implants may reduce the tinnitus perception [6]–[8] suggesting interplay between tinnitus and hearing impairment. In addition, a patient's reaction to tinnitus determines the degree of tinnitus-related distress which is largely independent from psychoacoustic measures [9], [10]. Besides that, especially those patients with high tinnitus-related distress show a high prevalence of depressivity, anxiety and somatic symptoms [11]–[14]. Depressivity and anxiety tend to worsen tinnitus-related distress and vice versa, but the relation between tinnitus and these psychopathologies is not undisputed [15].

Most therapeutic interventions focus on the reduction of tinnitus-related distress without primarily trying to reduce tinnitus loudness [16]–[18], partly reflecting the lack of successful approaches to reduce loudness. It should not be ignored, however, that the subjectively perceived loudness may be the major complaint, and that it may be associated with low tinnitus-related distress [19]. This together with the finding, that severe tinnitus-related distress may be associated with low subjective loudness [19] suggests that these measures represent separate tinnitus parameters that are both relevant to the affected individuals. They may independently become incapacitating and then demand specific therapeutic interventions.

The subjectively perceived loudness of tinnitus can be recorded by numeric rating scales which typically range from 0 or 1 (low loudness) to 10 (high loudness) and which have been used in several studies on tinnitus (e.g. [20], [21]). Numeric rating scales provide high measurement resolution and are easy to score [22], they reflect the subjective impression of tinnitus loudness experienced by the patients which may deviate from the tinnitus loudness that is measured by psychoacoustic matching procedures [23]. Tinnitus-related distress on the other hand is measured with psychometrically validated questionnaires (e.g. [24], [25]).

Aim of the present questionnaire-based study was to determine factors that differentially affect subjectively perceived tinnitus loudness and tinnitus-related distress. Data were collected from 4705 persons affected by tinnitus. The questionnaire included items about tinnitus characteristics such as localization and type of sound, tinnitus duration, subjectively perceived tinnitus loudness, and hearing impairment. Strength of tinnitus-related distress was recorded with the psychometrically validated short version of the Tinnitus Questionnaire (MTQ, [26]). Depressive symptoms, anxiety and somatic symptom severity were addressed with modules of the psychometrically validated Patient Health Questionnaire (PHQ, [27], [28]).

## Methods

### Data collection and sample

During September of 2010 a questionnaire with a total of 256 items was distributed by mail to all of the 13,349 registered patient members of the German Tinnitus Association (Deutsche Tinnitus-Liga, DTL). The questionnaire was accompanied by a letter in which the participants were informed, that by filling out and sending in the questionnaire they agreed to the use of their data for research purposes. 4752 questionnaires were received, and the data of 4705 questionnaires were entered into the data base. The rest was omitted mainly because of invalid membership numbers. The questionnaires were pseudonymized in that they contained the membership code but not the participants' names. The study protocol was approved by the local ethics committee (Ethikkommission II) of the Medical Faculty Mannheim of Heidelberg University and by the data safety commissioner of the Medical Faculty Mannheim of Heidelberg University according to the principles expressed in the Declaration of Helsinki.

Besides information about age and gender the following information included in the questionnaire was used for the present study:

### Tinnitus characteristics and hearing loss

In the questionnaire type of tinnitus sound, type of tinnitus onset (slowly/suddenly), its duration and localization as well as the time of daily tinnitus awareness was assessed. Subjectively perceived loudness was recorded on a numeric rating scale (T-NRS) from 0 (tinnitus audible only during silence) to 10 (louder than all external sounds). To asses the potential presence of hearing impairments it was asked, if an audiogram was taken, if hearing impairment had been diagnosed by an otolaryngologist, and if hearing aids were used. Response options were ‘yes’ or ‘no’. This did not accurately reflect the amount of hearing loss, but did tell whether hearing impairments were uni- or bilateral and which side was affected.

### Tinnitus-related distress

Tinnitus-related distress was addressed by the psychometrically validated brief version of the tinnitus questionnaire (MTQ: [26]) with sum scores from 0 (no distress) to 24 (maximum distress). Sum scores were derived only from cases with complete MTQ-scales. In line with Hiller and Goebel [26] sum scores below 8 were classified as mild tinnitus-related distress, while sum scores above 18 were seen as indicator of severe tinnitus-related distress.

### Psychological factors

Three psychometrically validated modules of the Patient Health Questionnaire (PHQ) were used to address depressivity (PHQ-9), anxiety (GAD-7), and somatic symptom severity (PHQ-15) [27], [28]. Response options for PHQ-9 and GAD-7 were 0 (not bothered at all) to 3 (bothered almost every day). Response options for PHQ-15 were 0 (not bothered at all) to 2 (bothered a lot). Higher scores indicated greater symptom severity in all scales, and a cut point at 15 distinguished between mild/moderate and severe/most severe symptom levels [28], [29]. A case was eliminated for classification in a module, if a single item in that module was missing. Since in the PHQ-15 one item addressed pre-menopausal women and one item addressed sexually active persons exclusively, these items were scored 0 if left blank producing a slight underestimation of somatic symptom severity in theses cases.

Finally, one question each asked about difficulties to initiate and to maintain sleep, and the presence of chronic somatic morbidities as well as chronic pain and dizziness were recorded.

### Data analysis

Data management and data analysis were performed with the Statistical Package for the Social Sciences (SPSS) 18.0 for Windows (Chicago, Illinois) and SAS for Windows 9.2 (SAS Institute Inc., Cary, NC, USA). Percentages are reported for categorical variables (% in table 1) and means ± standard deviations (mean [standard deviation]) for sum score and NRS variables. A correlation analysis for tinnitus-related distress (MTQ) and subjective loudness (T-NRS) showed a moderate correlation between them (Spearman's rho for non-parametric data: 0.524). Therefore, further analyses were performed separately for both tinnitus measures.

**Table 1:** Influence of Tinnitus characteristics, hearing impairments, and psychopathological factors on subjectively perceived tinnitus loudness and on tinnitus-related distress.

Characteristic %	Total	Subjective Tinnitus Loudness (T-NRS)			Tinnitus-Related Distress (MTQ)		
		Low	High	OR (95% CI)	Mild	Severe	OR (95% CI)
		(T-NRS≤2)	(T-NRS≥8)		(MTQ≤7)	(MTQ≥19)	
	N = 4705	N = 379	N = 1338		N = 1754	N = 623	
**Age**>50 years	74.9	**59.8**	**82.0**	**3.1 [2.4–3.9]**	73.0	75.6	1.1 [0.9–1.4]
**Female**	40.9	43.0	38.4	1.2 [1.0–1. 5]	39.7	36.8	1.1 [0.9–1.4]
**Time since tinnitus onset**							
< = 12 months	1.3	1.3	1.1	0.8 [0.3–2.2]	1.2	1.5	1.3 [0.6–2.9]
<12 months and < = 5 years	14.7	20.6	10.9	**0.5 [0.4–0.6]**	11.7	19.1	1.8 [1.4–2.3]
>5 years	84.0	78.1	88.0	**2.1 [1.5–2.8]**	87.1	79.4	0.6 [0.5–0.7]
**Tinnitus onset**							
sudden	66.2	69.9	67.1	0.9 [0.7–1.1]	65.8	66.9	1.1 [0.9–1.3]
slowly progressive	41.4	31.7	43.0	1.6 [1.3–2.1]	39.4	39.8	1.0 [0.8–1.2]
**Permanent awareness**	79.2	**49.3**	**92.3**	**13.6 [10.2–18.3]**	**70.9**	**91.6**	**5.4 [3.8–7.5]**
**Localization of the tinnitus**							
left	19.6	27.2	14.6	**0.5 [0.4–0.6]**	22.0	14.0	0.6 [0.5–0.7]
right	14.4	16.6	12.3	0.7 [0.5–1.0]	15.6	11.1	0.9 [0.7–1.2
binaural/central	74.2	**61.5**	**82.4**	**2.9 [2.3–3. 8]**	68.5	84.1	**2.4 [1.9–3.1]**
**Hearing impairment**							
unilateral left	18.3	20.8	17.4	0.8 [0.6–1.1]	18.2	18.2	1.0 [0.8–1.3]
unilateral right	13.6	11.1	12.0	1.1 [0.8–1.6]	13.7	12.8	0.9 [0.7–1.2]
bilateral	44.5	**26.6**	**56.7**	**3.6 [2.8–4.6]**	39.8	54.3	1.8 [1.5–2.2]
**Influence of Hearing aid**							
tinnitus lower	29.9	**36.5**	**22.2**	**0.5 [0.3–0.8]**	**39.6**	**16.0**	**0.3 [0.2–0.4]**
tinnitus louder	5.1	6.8	6.4	1.0 [0.4–2.5]	**2.6**	**10.8**	**4.6 [2.3–9.2]**
**Dizziness**	27.6	**17.9**	**33.0**	**2.3 [1.7–3.0]**	21.3	41.6	**2.6 [2.2–3.2]**
**Chronic pain**	66.2	**50.9**	**73.5**	**2.7 [2.1–3.4]**	**52.4**	**81.9**	**3.9 [3.1–4.9)**
**Somatic comorbidities**	53.7	**42.0**	**59.9**	**2.1 [1.6 –2.7]**	56.1	60.3	1.6 [1.3–2.0]
**Sleep Problems**	76.5	**60.4**	**83.9**	**3.4 [2.7–4.4]**	**60.9**	**94.8**	**11.6 [8.1–16.8]**
**Psychopathologies**							
Depressivity (PHQ-9≥15)	10.6	**5.3**	**20.0**	**4.5 [2.8–7.2]**	**1.5**	**42.6**	**48.0 [31.3–73.7]**
Anxiety (GAD-7≥15)	7.3	**3.0**	**14.0**	**5.3 [2.8–9.8**	**0.8**	**32.1**	**61.6 [34.8–109.2]**
Somatic Symptom Severity (PHQ-15≥15)	13.1	**3.5**	**22.5**	**8.0 [4.4–14.4]**	**2.9**	**39.8**	**22.1 [15.8–31.1]**

In this table, population percentages (%) as well as characteristics (%) of subgroups with low (< = 2) and high (≥8) subjectively perceived tinnitus loudness measured on a numeric rating scale (T-NRS) from 0 (tinnitus audible only during silence) to 10 (louder than all external sounds), and mild (MTQ score≤7) and severe (MTQ score≥19) tinnitus-related distress are shown. Absolute numbers deviate because of missing data in single items. Percentages for type of tinnitus onset and tinnitus localization exceed 100%, because participants with two distinguishable tinnitus tones coded multiple categories. Variables with values marked in bold because of odds ratios (OR) of 2 and above or 0.5 or below were included in the regression analysis.

CI – confidence interval of OR, MTQ – short version of the tinnitus questionnaire, N – number.

To identify the variables with the strongest association with MTQ and T-NRS respectively, groups with mild (MTQ-score≤7) versus severe (MTQ-score≥19) tinnitus-related distress, and with low (T-NRS≤2) versus high loudness (T-NRS≥8) were compared. Conceivably loudness and distress clusters do partially overlap. Data were categorized into major (problematic) versus minor with a cut-off score of ≥15 (major) for the variables PHQ-9, GAD-7 and PHQ-15 distinguishing severe levels of depressivity, anxiety and somatic symptom severity [28], [29]. Based on this grouping odds ratios were computed. The presence (major) of hearing impairment, dizziness, chronic pain, somatic comorbidities, and sleep problems were contrasted to their absence (minor). In addition, the tinnitus characteristics sudden onset, constant awareness of the tinnitus, and localization were included. An odds ratio (OR) of 2 and above respectively of 0.5 and below, indicating a 2 (or more)-fold likelihood respectively a 0.5 (or less)-fold likelihood of a characteristic were considered relevant. In addition to the point estimates, 95% confidence intervals were calculated for the odds ratios to quantify the range of the effect size. The predictive power on T-NRS or MTQ of variables with an OR≥2 or ≤0.5 in the univariate analyses was assessed in a binary stepwise logistic regression model.

## Results

Data were derived from 4705 questionnaires. The age range was 18 to 94 years (58.63 [11.76] years; females: 57.44 [12.22]; males: 59.47 [11.39]), and the overall female proportion was 40.9%. Mild tinnitus-related distress (MTQ-score≤7) was reported by 37.6%, whereas distress related to the tinnitus was judged as being intermediate (8≤MTQ-score≤18) by 49.0%, and 13.4% of the participants felt severely distressed by their tinnitus (MTQ-score≥19). Low subjective loudness was commonly associated with mild distress, whereas high subjective loudness tended to be associated with severe distress. These categories were not congruent, however. In 13 participants (0.3%) a subjective loudness score (T-NRS) of two and lower combined with severe tinnitus–related distress, whereas 209 participants (4.4%) reported a combination of loud (T-NRS≥8) but only mildly distressing tinnitus. Overall, the correlation between T-NRS and MTQ was found to be moderate (r = 0.524 Spearman's rho based on MTQ sum scores). Gender effects were not apparent, whereas older age and tinnitus duration of more than 5 years were associated with louder but not with a more distressing tinnitus. Sudden onset was prevalent, and of the 50% that named a possible cause, the majority (n = 1716) suspected stress followed by sudden hearing loss (1389) and noise trauma (354) as putative reasons. Ringing, continuous, binaural-central tinnitus prevailed, and unilateral tinnitus was more frequently located on the left ear which coincided with a higher incidence of left unilateral hearing impairment (table 1). The subjectively perceived loudness had increased since tinnitus onset in 34.9% of the participants while a decrease was reported by 7.7%. In contrast, decreases in tinnitus-related distress (28.4%) were as common as increases (25.4%).

### Auditory-related factors

Altogether 78.1% reported a hearing impairment that had been diagnosed audiometrically by an otolaryngologist. This percentage rose with age (56.2% below age 40; 84.1% above age 70). Percentages of bilateral hearing impairment, binaural-central localization and permanent awareness of the tinnitus differed substantially when comparing low and high subjective tinnitus loudness (table 1).

42% of the individuals indicating hearing impairments used hearing aids. 29.9% of them experienced a decrease while 5.1% experienced an increase in the subjective loudness of the tinnitus when using hearing aids. Participants with low subjective loudness (T-NRS≤2) as well as participants with mild distress (MTQ≤7) benefitted most from the use of hearing aids, whereas those with severe tinnitus-related distress (MTQ≥19) reported loudness increases most often and decreases least often (table 1).

### Psychological factors

**Figure 1:**
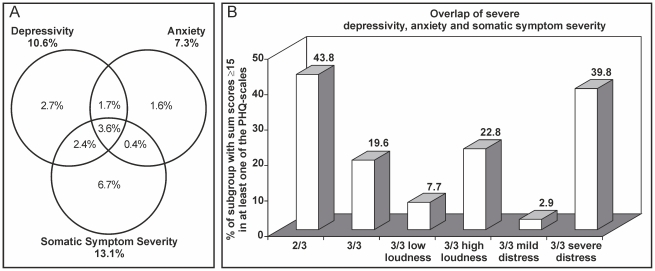
Comorbidity of depressivity, anxiety, and somatic symptom severity derived from scores of the Patient Health Questionnaire (PHQ). **A**: Somatic symptom severity was most common (13.1% of whole sample) followed by depressivity (10.6%) and anxiety (7.3%). 3.6% of the whole sample were affected by elevated levels of depressivity, anxiety and somatic symptom severity at the same time, and an additional 4.5% showed elevated levels in two scales. **B**: The 726 participants with scores of 15 or above in at least one of the three PHQ scales (1/3) were set to 100%. Of these, 318 (43.8%) exhibited severe levels in at least 2 PHQ-scales (2/3), while 142 (19.6%) had severe levels in all scales (3/3). The percentage of participants with scores of 15 and above in all three scales (3/3) was least common in the subgroup with mild tinnitus-related distress (2.9%), while it was most common in the subgroup with severe tinnitus-related distress with 39.8%. Differences between subgroups with low and high subjective loudness had the same direction, but were less pronounced. PHQ scales: PHQ-9 – depressivity, GAD-7 – anxiety, PHQ-15 – somatic symptom severity.

Correlations between depressivity, anxiety, somatic symptom severity and T-NRS were low (Spearman's Rho: PHQ-9: 0.351; GAD-7: 0.301; PHQ-15: 0.312). Correlations between MTQ and the PHQ-scales (PHQ-9: 0.663; GAD-7: 0.610; PHQ-15: 0.535) exceeded those between MTQ and T-NRS (0.524). Most noteworthy was the high incidence of elevated PHQ scores in the group with severe tinnitus-related distress (table 1). Correlations between the three PHQ scales exceeded all other correlations (PHQ-9/GAD7: 0.805; PHQ-9/PHQ-15: 0.762; GAD-7/PHQ-15: 0.654). Altogether 726 participants, equalling 18.6% of the whole sample, reached a score of 15 and above in at least one of the PHQ scales. Severe somatic symptom severity was most common (13.1% of whole sample) followed by depressivity (10.4%) and anxiety (7.3%), and 3.6% expressed severe levels in all PHQ-scales. Based on the population with a severe level in at least one of the PHQ scales (726 = 100%), the percentage with comorbid severe depressivity, anxiety and somatic symptom severity was highest in the subgroup with severe tinnitus-related distress (39.8%), and lowest in the subgroup with mild tinnitus-related distress (2.9%; Fig. 1).

### Influence of somatic comorbidities and sleep quality

More than 50% of the sample reported somatic comorbidities. Percentages were lower only in the group with low subjective loudness. Reports of sleep disturbances, chronic pain and dizziness peaked in the group with severe tinnitus-related distress. Analysis of OR revealed that sleep disturbances, chronic pain and dizziness were associated with tinnitus-related distress, and that all variables were associated with subjective loudness although to a lesser extent (table 1).

### Differentiation between subjective tinnitus loudness and tinnitus-related distress

Univariate analyses showed that groups with low and high subjective tinnitus loudness differed substantially (OR≥2 or ≤0.5) with respect to the percentage of subjects that was permanently aware of the tinnitus, somatic symptom severity, binaural/central localization of the tinnitus, anxiety and depressivity, binaural hearing impairment, tinnitus duration, and in the variables sleep problems, age, chronic pain, dizziness, and somatic comorbidities. Dominance of loudness over distress was most obvious for those with loud but only mildly distressing tinnitus (n = 209). In this group the low sum scores in the PHQ scales (PHQ-9: 4.0 [3.6], GAD-7: 3.2 [2.9]; PHQ-15: 5.7 [4.0]) were most notable.

In contrast, groups with mild respectively severe tinnitus-related distress differed most in the percentage of elevated depressivity, anxiety, somatic symptom severity, and sleep disturbances. Permanent awareness and localization of the tinnitus as well as chronic pain and dizziness were less influential, whereas bilateral hearing impairment age and tinnitus duration had no substantial influence.

The predominant characteristic in the group with severe tinnitus-related distress was the high prevalence of psychologically relevant symptoms as indicated by elevated scores in the PHQ scales. Conspicuous were also the high percentages of those with comorbid chronic pain and dizziness. Moreover, the majority had difficulties in initiating or maintaining sleep, and overall benefit from hearing aids was lower than average. Influence of the psychopathological variables was most obvious in a small cluster of 13 subjects who reported low subjective tinnitus loudness but severe tinnitus-related distress. Mean sum scores of PHQ-9 (13.3 [5.0]), GAD-7 [12.2 [6.5]) and PHQ-15 (15.2 [9.0] were high, whereas hearing impairments (69.2%; bilateral: 46.2%) were less frequent than average.

In a step-wise regression analysis the predictive power of auditory and non-auditory variables on tinnitus-related distress respectively subjective tinnitus loudness with the factors showing OR of 2 and above or of 0.5 and below was calculated. Factors found to be relevant for subjective tinnitus loudness was above all the factor “permanent awareness” of the tinnitus followed by “binaural/central localization”, sleep problems and pain. In contrast, most influential variables on tinnitus-related distress were depressivity and anxiety followed by sleep problems and permanent awareness of the tinnitus. In addition the variables binaural/central tinnitus localization as well as somatic symptom severity and pain had significant influence (table 2). Noteworthy was also that decreases of subjective tinnitus loudness while using a hearing aid were significantly more likely for those with mild tinnitus-related distress than for those with severe tinnitus-related distress as indicated by an OR below 0.5 (table 1, 2).

**Table 2:** Results of the stepwise regression analysis.

Tinnitus-related distress (MTQ)	OR [95% CI]	Subjective tinnitus loudness (T-NRS)	OR [95% CI]
Depressivity (PHQ-9)****	19.67 [5.29–73.12]	Permanent awareness of tinnitus****	24.04 [9.25–62.45]
Anxiety (GAD-7)*	14.19 [1.52–132.42]	Binaural tinnitus localization***	4.77 [2.00–11.37]
Sleep problems****	11.99 [3.46–41.51]	Sleep Problems**	3.23 [1.40–7.49]
Permanent awareness of tinnitus***	10.61 [2.43–46.29]	Pain*	2.84 [1.20–6.73]
Binaural tinnitus localization**	4.14 [1.62–10.57]		
Somatic Symptom Severity (PHQ-15)*	4.75 [2.43–46.29]		
Pain*	2.23 [1.14–4.38]		
Tinnitus lower with hearing aid*	0.32 [0.15–0.7]		

Variables with significant impact on tinnitus-related distress and subjectively perceived tinnitus loudness were determined in a stepwise regression analysis comparing mild versus severe distress and low versus high loudness, respectively. Concordance of the model was 89.7 for tinnitus-related distress and 81.3 for subjective tinnitus loudness. Odds ratios (ORs) and 95% confidence intervals (95% CI) are shown.

* p<0.05,

** p<0.01,

*** p<0.001,

**** p<0.0001.

MTQ – short version of the tinnitus questionnaire, T-NRS – numeric rating scale for subjectively perceived tinnitus loudness.

## Discussion

The results of the present study are based on data obtained from 4705 participants with tinnitus. The observed distribution of tinnitus characteristics is in accordance with those found in epidemiological tinnitus studies [21], [26], [30]. The results show that subjective tinnitus loudness and tinnitus-related distress are only moderately correlated, and that the variables that exert a major influence on either tinnitus measure differ substantially. Bilateral hearing impairment rather increases the risk for a tinnitus that is perceived as being loud, whereas variables associated with psychopathologies rather increase the risk for severe tinnitus-related distress. Therefore subjective tinnitus loudness should be treated as a separate characteristic of the tinnitus in addition to tinnitus-related distress. These findings are in line with earlier reports [19], [26], [31], and with imaging studies that suggest involvement of different brain areas in the processing of the tinnitus percept compared to tinnitus-related distress, i.e. the reaction on this percept (rev. in [13]). Moreover they can be seen as an explanation for the finding that therapies like cognitive behavioural therapy which aim at the reaction on the tinnitus, do not influence its perception, i.e. the subjectively perceived loudness [16]–[18]. In addition, there is evidence that the subjectively perceived loudness can be diminished temporarily while not influencing tinnitus-related distress by electrically stimulating dorsolateral prefrontal cortex [20].

### Tinnitus related distress: the influence of psychopathologic variables

To our knowledge this study analyzes the largest sample of subjects with tinnitus that has ever been evaluated with psychometrically validated questionnaires addressing depressivity, anxiety and somatic symptom severity in conjunction with subjective tinnitus loudness and tinnitus-related distress. The observed percentages of severe depressivity and anxiety are slightly higher than in the general population [32]. In the subgroup with severely distressing tinnitus (MTQ>18), however, this percentage is multiplied, which is in accordance with reports on a close association of a severely distressing tinnitus with depressive symptoms and anxiety [2], [12], [14], [33], [34].

In addition, severe somatic symptom severity is increased in the group with severe tinnitus-related distress compared to the study population in general. The threshold we used for the distinction between moderate and severe cases requires the presence of at least seven bothering somatic symptoms, and is seen as a reliable distinction between presence of somatoform disorders in comparison to their absence [28]. The present finding also is in line with observations that tinnitus patients with high levels of self- and somatic attention express greater emotional and tinnitus-related distress [35]–[37], and that depressed tinnitus patients display strong somatic focus resulting in a tendency to report large numbers of medically unexplained symptoms [36].

In agreement with former tinnitus studies [14], [35], [36], somatic symptom severity as a determining factor for tinnitus-related distress is most frequent followed by depressivity, while anxiety is least frequent in the present sample. Although depressivity, anxiety, and somatic symptom severity are often coexistant, there is no complete overlap in the study sample. Moreover relative frequencies deviate between the subgroups with low and high subjective loudness as well as between subgroups with mild and severe tinnitus-related distress, and they deviate from those seen in a large primary care population [28].

An important issue that cannot be settled with this cross-sectional survey is whether these comorbidities are primary or secondary to the tinnitus. Longitudinal studies with a small number of acute tinnitus patients suggest that psychopathological conditions exist beforehand and constitute risk factors for the development of a distressing tinnitus or that they arise together with the tinnitus [33], [37]. This does not exclude the possibility, however, that tinnitus promotes the progression of psychopathologies and it appears likely that both developments exist.

### Variables that predominantly influence the subjectively perceived tinnitus loudness and the effect of hearing aids

The rate of hearing impairment in the present study is high. Its incidence increases with age, and bilateral hearing impairment is more frequent in the group that experiences loud tinnitus. These findings agree with those of others [3], [38]–[40]. A relation between the amount of hearing loss and subjective tinnitus loudness was reported by two studies comprising together about 1000 audiometrically screened tinnitus patients indicating that tinnitus loudness correlates with the presence and the degree of threshold shifts [39], [40].

Hearing impairment is thought to be the permissive condition for the development of the tinnitus perception. Therefore restoring auditory input is expected to reduce the subjectively perceived tinnitus loudness. Though, results of such interventions are variable and the overall success rate is low [6], [41]. The results of the present study indicate that recovery of auditory input reduces subjective tinnitus loudness while using the hearing aid in about 30% of all hearing aid users. Most important, however, the results suggest that hearing aids are more effective in individuals that have a non-distressing tinnitus, whereas the risk to increase the perceived tinnitus loudness when using hearing aids is disproportionately high in individuals with severe tinnitus-related distress.

### Limitations

Some limitations apply to the present analysis. Our tinnitus population may not represent the tinnitus population as a whole, but may be dominated by individuals that are concerned by their tinnitus and became active by joining a patient organization. Therefore the reported characteristics may not be entirely representative for the general tinnitus population. Furthermore the evidence relies on self-report questionnaires and therefore may be influenced by misconceptions. However, characteristics of the investigated tinnitus population are in line with the published literature [11], [14], [21], [30], [34].

### Conclusion

Subjectively perceived tinnitus loudness as measured here represents a distinctive quality of tinnitus which needs to be assessed separately and in addition to tinnitus-related distress and to psychoacoustic tinnitus characteristics. This can be done effectively by numeric rating scales. Study participants with a severely distressing tinnitus expressed elevated levels of depressivity, anxiety, and somatic symptom severity, and the high incidence of sleep problems, chronic pain and dizziness in the highly distressed tinnitus patients appears to be associated foremost with these factors. Therefore, especially in subjects with high tinnitus-related distress, the potential presence of severe depressivity, anxiety and somatic symptom severity should be assessed separately from tinnitus-related distress by validated psychopathology questionnaires.

A combination of the MTQ questionnaire with established questionnaires addressing psychopathologies such as PHQ-9, GAD-7, and PHQ-15 in conjunction with audiological examination and recording of the subjectively perceived tinnitus loudness represents a powerful and easy to handle tool to characterize tinnitus patients. While these parameters are already assessed in specialized tinnitus centres, they also need to be evaluated during routine otolaryngologic examination of tinnitus patients. As suggested by the differential effect of hearing aids in distressed and non-distressed participants, a comprehensive characterization may optimize patient allocation and consequently the therapeutic outcome of existing tinnitus therapies.
