# Correction: Tinnitus: Distinguishing between Subjectively Perceived Loudness and Tinnitus-Related Distress

**DOI:** 10.1371/annotation/96f457f9-3f48-4f88-a7f0-1d5e6067e7a5

**Published:** 2012-09-05

**Authors:** Elisabeth Wallhäusser-Franke, Joachim Brade, Tobias Balkenhol, Roberto D'Amelio, Andrea Seegmüller, Wolfgang Delb

There were errors in the published funding and competing interest statements. The correct statements are:

Funding: This work was partly supported by the German Tinnitus Association (DTL), auric Hörsysteme and Schaaf und Maier Hörgeräte. The funders had no role in study design, data collection and analysis, decision to publish, or preparation of the manuscript.

Competing interests: This work was partly supported by the German Tinnitus Association (DTL), auric Hörsysteme and Schaaf und Maier Hörgeräte. This does not alter the author's adherence to all the PLoS One policies on sharing data and materials. 

